# Assistive HCI-Serious Games Co-design Insights: The Case Study of i-PROGNOSIS Personalized Game Suite for Parkinson’s Disease

**DOI:** 10.3389/fpsyg.2020.612835

**Published:** 2021-01-15

**Authors:** Sofia Balula Dias, José Alves Diniz, Evdokimos Konstantinidis, Theodore Savvidis, Vicky Zilidou, Panagiotis D. Bamidis, Athina Grammatikopoulou, Kosmas Dimitropoulos, Nikos Grammalidis, Hagen Jaeger, Michael Stadtschnitzer, Hugo Silva, Gonçalo Telo, Ioannis Ioakeimidis, George Ntakakis, Fotis Karayiannis, Estelle Huchet, Vera Hoermann, Konstantinos Filis, Elina Theodoropoulou, George Lyberopoulos, Konstantinos Kyritsis, Alexandros Papadopoulos, Anastasios Depoulos, Dhaval Trivedi, Ray K. Chaudhuri, Lisa Klingelhoefer, Heinz Reichmann, Sevasti Bostantzopoulou, Zoe Katsarou, Dimitrios Iakovakis, Stelios Hadjidimitriou, Vasileios Charisis, George Apostolidis, Leontios J. Hadjileontiadis

**Affiliations:** ^1^Faculdade de Motricidade Humana, Centro Interdisciplinar de Performance Humana, Universidade de Lisboa, Lisbon, Portugal; ^2^Lab of Medical Physics, Aristotle University of Thessaloniki, Thessaloniki, Greece; ^3^Centre for Research and Technology Hellas, Information Technologies Institute, Thessaloniki, Greece; ^4^Fraunhofer Institute Intelligent Analysis and Information Systems, Sankt Augustin, Germany; ^5^PLUX, Wireless Biosignals, Lisbon, Portugal; ^6^Karolinska Institutet, Solna, Sweden; ^7^Elliniko Kentro Kenotomias Microsoft, Athens, Greece; ^8^AGE Platform Europe, Woluwe-Saint-Pierre, Belgium; ^9^COSMOTE Kinites Tilepekoinonies AE, Athens, Greece; ^10^Multimedia Understanding Group, Information Processing Laboratory, Department of Electrical and Computer Engineering, Aristotle University of Thessaloniki, Thessaloniki, Greece; ^11^International Parkinson Excellence Research Centre, King’s College Hospital NHS Foundation Trust, London, United Kingdom; ^12^Department of Neurology, Technical University Dresden, Dresden, Germany; ^13^Third Neurological Clinic, G. Papanikolaou Hospital, Thessaloniki, Greece; ^14^Department of Electrical and Computer Engineering, Aristotle University of Thessaloniki, Thessaloniki, Greece; ^15^Department of Electrical Engineering and Computer Science/Biomedical Engineering, Khalifa University of Science and Technology, Abu Dhabi, United Arab Emirates

**Keywords:** human-computer interaction-serious games, co-creation, game-based learning, i-PROGNOSIS, Parkinson’s disease

## Abstract

Human-Computer Interaction (HCI) and games set a new domain in understanding people’s motivations in gaming, behavioral implications of game play, game adaptation to player preferences and needs for increased engaging experiences in the context of HCI serious games (HCI-SGs). When the latter relate with people’s health status, they can become a part of their daily life as assistive health status monitoring/enhancement systems. Co-designing HCI-SGs can be seen as a combination of art and science that involves a meticulous collaborative process. The design elements in assistive HCI-SGs for Parkinson’s Disease (PD) patients, in particular, are explored in the present work. Within this context, the Game-Based Learning (GBL) design framework is adopted here and its main game-design parameters are explored for the Exergames, Dietarygames, Emotional games, Handwriting games, and Voice games design, drawn from the PD-related i-PROGNOSIS Personalized Game Suite (PGS) (www.i-prognosis.eu) holistic approach. Two main data sources were involved in the study. In particular, the first one includes qualitative data from semi-structured interviews, involving 10 PD patients and four clinicians in the co-creation process of the game design, whereas the second one relates with data from an online questionnaire addressed by 104 participants spanning the whole related spectrum, i.e., PD patients, physicians, software/game developers. Linear regression analysis was employed to identify an adapted GBL framework with the most significant game-design parameters, which efficiently predict the transferability of the PGS beneficial effect to real-life, addressing functional PD symptoms. The findings of this work can assist HCI-SG designers for designing PD-related HCI-SGs, as the most significant game-design factors were identified, in terms of adding value to the role of HCI-SGs in increasing PD patients’ quality of life, optimizing the interaction with personalized HCI-SGs and, hence, fostering a collaborative human-computer symbiosis.

## Introduction

For the first time, a Serious Game (SG) became available by prescription in the United States, seen as a first-ever U.S. Food and Drug Administration (FDA)-approved digital treatment that builds on a tradition of gaming as a therapeutic tool that extends back more than a decade (Anderson, 2020). Clearly, the concept of a computer game encapsulates the Human-Computer Interaction (HCI) element, that plays a critical role in the study of games, as users are involved in game input/outcome mechanisms and play experiences. In this line, HCI-SGs can be transferable to many fields, such as education, rehabilitation, training, as they potentiate the re-education of the end-users (Michael and Chen, 2005). SGs, viewed here similarly to Michael and Chen (2005), are set in an assistive HCI-SGs symbiotic environment for Parkinson’s Disease (PD) patients, and their design elements are evaluated on positively affecting the PD patients. In the last decades, researchers have developed several non-invasive, objective methods for detecting early symptoms of PD by using physiological biomarkers, including techniques based on 3D motion analysis (using depth sensors) and techniques to examine motion signals (using wearable sensors) (Patel et al., 2009; Cancela et al., 2011; Stawarz et al., 2012; Procházka et al., 2015). In this line, personalization has become vital in game-based learning perspective, in order to optimize user/player experience and performance. These advances in sensor technology allow to acquire physiological data of users/players, permitting for the acquisition of more fine-grained information on internal changes of the player than conventional user interaction data (Ninaus et al., 2019). HCI-SGs that have been used to tackle main PD symptoms have been explored in several areas, i.e., (a) exercise-based SGs (ExerGames): mechanics of gait (Ekker et al., 2016), presence of tremor (Szlufik et al., 2016), bradykinesia and limited range of motion (Paraskevopoulos et al., 2014), balance and coordination issues (Konstantinidis et al., 2016), abnormal posture and physical status (Abbruzzese et al., 2016); cognitive and physical/motor rehabilitation (Barry et al., 2014; Garcia-Agundez et al., 2019; Garcia-Agundez, 2020); (b) dietary habits-based SGs (DietaryGames): mechanics of meal (Argolo et al., 2013), daily meal distribution (Blackburne et al., 2016), preferred food characteristics (Papapanagiotou et al., 2015), dietary quality (Dias et al., 2016b); (c) emotional aspects-based SGs (EmoGames): non-motor symptoms and mental health (Wang et al., 2013), and hypomimia (Vinokurov et al., 2015); and (d) evidence for handwriting-voice aspects-based SGs (Handwriting-Voice (H/V) Games): handwriting (Liu et al., 2013) and voice mechanics (Lv et al., 2015). A thorough description on the aforementioned aspects can be found in Dias et al. (2017a).

Personalized Game Suite (PGS) of the H2020 i-PROGNOSIS project (see footnote) is used here as the placeholder of the analyzed HCI-SGs. The motivation behind the PGS is to mitigate the PD symptoms using a gamified environment based on a personalized approach that involves different HCI-SGs, i.e., ExerGames, DietaryGames, EmoGames, and Handwriting/Voice Games, all integrated under a unified platform.

## Background

### PGS Design Targeting PD Symptoms

**FIGURE 1:**
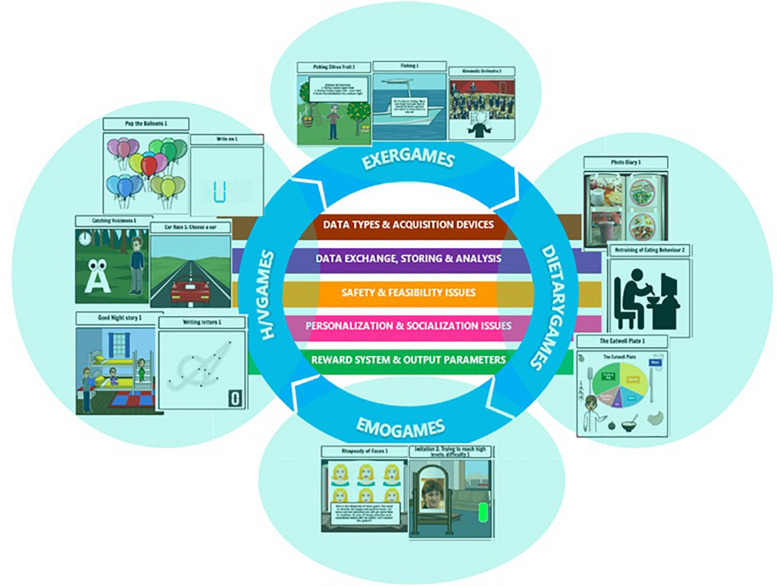
The i-PROGNOSIS Personalized Game Suite (PGS) conceptual framework, including 14 different games (i.e., three ExerGames. three DietaryGames. two EmoGames, and six Handwriting/Voice Games) based on five key transversal aspects, namely: (i) Data types and acquisition devices; (ii) Data exchange, storing and analysis; (iii) Safety and feasibility’ issues; and (iv) Personalization and socialization issues; and (v) Reward system and output parameters.

As described in Dias et al. (2017b), an underlying framework is adopted in the i-PROGNOSIS PGS for the design of each game, based on five key transversal aspects (see Figure 1), including: (1) Types of data and acquisition means, such as depth cameras, touch screens and tablets; (2) Exchange of data, storing and analysis, such as metrics during the game (in-game metrics), frequency of playing the game; (3) Issues regarding safety and feasibility, such as any possible feedback to avoid injuries; (4) Issues referring to personalization and socialization, such as, how much adaptive are the HCI-SGs to PD patients’ performance; potentialities for group-based playing to promote emulation among the patients; and (5) Systems that provide rewarding and output parameters, such as specific messages that serve as rewards and/or motivational triggers to increase the engagement of the users during the game). More detailed information (including the description of the 14 PGS game-scenarios) can be found in work of Dias et al. (2017b). Taking into account the early stage PD patients’ needs and requirements, the PGS aims to integrate different HCI-SGs in a unified platform, targeting, in a holistic way, PD symptoms (motor and non-motor) by providing a set of games (i.e., ExerGames, DietaryGames, EmoGames, and Handwriting/Voice Games) that address such symptoms within a gamified approach (Dias et al., 2016a; Hadjidimitriou et al., 2016) (see Table 1). Hence, these HCI-SGs are embedded within a common design context, namely Game-Based Learning (GBL), exhibiting inter-dependence and inter-functionality, as depicted in Figure 1.

**TABLE 1:** The correspondence of the game type to PD targeted symptomatology.

Game type	PD targeted symptomatology
ExerGames	Walk movement, gait freeze, presence of tremor, bradykinesia, rigidity, limited range of motion, balance and coordination, abnormal posture
DietaryGames	Dysphagia, compulsive eating behavior, anosmia, constipation
EmoGames	Depression, hypomimia, loss of self-confidence, feelings of shame, anxiety, deterioration of body image, embarrassment and stress, social isolation, decreased blink rate
Handwriting/VoiceGames	Micrographia, dysarthria, dysphonia

### PGS Common Conceptual Framework

GBL has a long tradition in education field; however, a formal introduction of GBL by the Technology Enhanced Learning community appeared in the work of Prensky (2001). The GBL approach, as a part of entertainment education, it intends to make learning enjoyable (Breuer and Bente, 2010). It can include the use of any type of games, for instance: board games, card games, digital games, or Exergames. Furthermore, well-designed GBL applications can immerse users into virtual environments that have a familiar and relevant look and feel. However, when GBL is placed on an effective context, the focus is placed on the experience the consequences that derive from the user’s actions and choices. Clearly, the possibility to make mistakes as s/he experiments the learning context, fosters active learning and practicing behaviors that can easily be transferred from the gamified environment into real life (Chatziantoniou and Politopoulos, 2018).

GBL can be met as adopted design model in various contexts; some examples include: GBL for Older Adults (gambaloa project) (Charlier et al., 2012), as an EU Lifelong Learning Programme that explored the effectiveness of using games with older adult learners placed within a variety of learning settings [(non)/(in)formal), including both (under)graduate curricula in higher education institutions and adult learning (learners aged >50 years, mixed with younger learners (>25years)], with potentialities to be learnt and applied more universally (Charlier et al., 2012).

The adoption of GBL within a vocational educational/training platform was recently reported in the Kotsifakos et al. (2018). In the latter, by spurring GBL, an effort was placed to surpass the traditional learning approaches. Moreover, GBL pattern was employed in the design of Internet-of-Things environments (Mavroudi et al., 2018). A multifactorial analysis of the important game-design factors, such as game mechanism/goals/value/narrative/mystery/challenges/sociality, from a macro-design perspective was presented by Shi and Shih (2015). Their goal was to support game designers in the way they design and combine game elements. In this vein, they proposed 11 basic factors that their relationships play important role in a successful GBL design model and validated the latter via two examples, justifying the positive effect of GBL design model in designing interesting HCI-SGs (Shi and Shih, 2015).

### Research Questions

Based on the aforementioned promising characteristics of the GBL design model, its main game-design parameters are adopted here and combined with all games included in the i-PROGNOSIS PGS framework, stating the following research questions (RQ1-RQ3):

• RQ1: What are the most important GBL-based game design factors that support effective HCI-SGs design for PD patients?
• RQ2: What are the necessary adaptations that should be applied to the GBL framework, in order to maximize the transferability of the PGS beneficial effect to real-life, taking into consideration real-life functional PD symptoms?
• RQ3: What are the necessary guidelines that game designers should follow when they intend to design ExerGames, DietaryGames, EmoGames, and Handwriting/VoiceGames in PD contexts?

To address the aforementioned RQs, the methodological steps, design factors, data acquisition settings, and analysis approaches that are described in the succeeding sections were followed.

## Methodology

### Methodological Steps of the Study

A six-step methodological plan within the PGS framework was adopted to address the above listed RQs. More specifically:

• Step 1: By employing the storyboard tool^1^, PD patients-tailored HCI-SGs scenarios were structured, in order to better visualize and illustrate (in a sequence of images) the main structural elements of the SG scenarios. In fact, seen as hierarchically structured sequence of graphs, the Storyboarding technique has been revealed as common technique in the HCI field and design, for facilitating the demonstration of system interfaces and contexts of use (Truong et al., 2006). Analytical description of all storyboards can be found in Dias et al. (2017b).
• Step 2: Based on the model proposed by Shi and Shih (2015), a selection of the GBL characteristics that express the design attributes of each SGs was carried out, focusing at the core elements needed to construct the game-design (see section “GBL-Based Selected Game-Design Factors”).
• Step 3: Exploration of aspects of the “co-creation” approach related with the design of the storyboards, to assist the preparation and feasibility of a larger investigation (Step 4) (see section “Co-creation Approach”) (see also Savvidis et al., 2018; Supplementary Figure 1).
• Step 4: Construction of a Web-survey (see section “Co-creation Approach”) that combines the outcomes from Steps 1 to 3, to be disseminated to different potential stakeholders of the PGS, empowering them as “co-creators” and active participants in the design process (see Supplementary Figure 2).
• Step 5: Application of linear regression analysis (Montgomery et al., 2012) on selected data from Step 4 (with focus on ExerGames category only). This analysis provides the means to evaluate the way each used game-design factor of Step 2 contributes to the whole game experience, leading to the introduction of a regression equation with the most significant ones, in terms of increased transferability of the HCI-SG context to real-life scenarios (Dias et al., 2018).
• Step 6: Application of linear regression analysis to additional data from Step 4 covering all categories of the games (i.e., ExerGames, DietaryGames, EmoGames, Handwriting/VoiceGames), showing the capability to predict the transferability of the PGS to real-life scenarios, addressing many functional PD symptoms and maximizing the positive effect of the integrated HCI-SGs.

### GBL-Based Selected Game-Design Factors

Based on established theories/perspectives, such as behaviorism, constructivism, cognitivism, psychology, HCI-SGs design factors can be selected in order to better support user’s active learning, engagement and critical thinking within the game (Gee, 2007). Here, the GBL-based design model proposed by Shi and Shih (2015), according to Step 2 (see section “Methodological Steps of the Study”), was adopted, focusing at the core design factors listed below:

• Game goals,
• Game rules and gameplay,
• Game plot/story,
• Game options,
• Levels of challenge,
• Game surprises,
• Game causalities, and
• Transfer into real life.

These design factors are essential to the game structure and provided the common basis across all HCI-SGs, in order to identify the most significant ones per HCI-SG (see section “Results and Discussion”) that efficiently predict the transferability of the HCI-SGs to real-life context, targeting the related PD symptoms (see Table 1). Moreover, these design factors are main parameters in satisfying the main criteria for designing high-quality serious games proposed by Caserman et al. (2020), derived from game-related literature. Based on the adopted design factors, the balance between game and serious aspects is considered, by securing clear goals, following game rules and gameplay with suitable feedback, within an enjoyable game plot/story and adequate game options, including various levels of challenges and surprises to ensure the user engagement and adapted game flow (considering the users’ skills). The adopted game causalities could further support the inclusion of the game goals within the gameplay, in a way that better links the serious with the game part during playing the game.

### Co-creation Approach

Overall, “co-creation” game approaches are based on the concept that users’ opinions are essential in the creative process, since the users provide insight into what is valuable to them; meaning that the co-creation process can be any process that brings together users and game designers to work toward a shared goal. Apparently, this is an important factor in the way the HCI aspect is optimally integrated within the design of the SGs. In practice, this concept takes the form of a collaborative work in which stakeholders, researchers, designers, and end-users explore a problem and produce solutions together, considering their different approaches, needs, and perspectives. In this way, different types of stakeholders in HCI-SGs design settings are involved; an extremely important aspect (Emmerich and Bockholt, 2016). Within a “co-creation” environment, different opinions, experiences and ideas are productively blended, allowing for interaction and collaboration amongst, for example, game researchers and users, resulting in further understanding of the role of game design elements in the maximization of the player’s experience and game impact in their life. In the same vein, interaction of the users with developers and researchers can help them to identify successful and failed game concepts, assisting them improving the effectiveness in their design of HCI-SGs (Emmerich and Bockholt, 2016). From these perspectives, a triangulation strategy based on different kind of sources (i.e., literature review, users’ perspective, medical opinion) were considered to readjust and consequently improve the 14 constructed game scenarios of the PGS.

### Web-Survey

Step 4 (see section “Methodological Steps of the Study”) was realized using a specifically constructed Web-survey, that underwent initial review by experts in the field toward its optimized version in terms of redundancy and time completion minimization and information acquisition maximization (see Supplementary Figure 2). The Web-survey was designed to reveal the role of the eight factors derived from Step 2 and 18 questions, in total, were employed, accordingly, combining both closed, with unique response within a 5-point Likert-type scale (1-strongly disagree; 5-strongly agree), and open questions. The Web-survey followed the organization described below:

• Part I: This is an introductory part to the participant that initially presents an epitomized version of the i-PROGNOSIS PGS approach and a set of general instructions. Then, participant’s demographic information (such as age, gender) is gathered, followed by his/her specific role, i.e., if s/he is PD patient, PD-expert healthcare, Researcher, Game designer, and PD-free Participant.
• Part II: This part relates with more general questions based on: (a) the use of technological artifacts, such as smartphone, tablet, smart TV, (b) the user’s preferences on the SGs from the PGS, in terms of usefulness and contribution in: prevention, mitigating PD symptoms, monitoring the health status, entertainment, and (c) user’s prior experience on SGs.
• Part III: This part initially involves a visual information with a short video (1 min) per SG scenario, based on animated version of the storyboards from Step 1. Then, each of the eight game design factors (see section “GBL-based Selected Game-Design Factors”) is briefly described and its adaptation to the characteristics of each game-scenario follows, leading to related questions to capture the responder’s impact of each game design factor.
• Part IV: This part addresses PD specialists only, including additional (open) questions to connect the whole structure and characteristics of the game with the PD-related symptoms.

The completion of the Web-survey for the whole set (14) of PGS games scenarios lasted, on average, 40 min per participant.

### Experimental Setup and Implementation Issues

#### Semi-Structure Interviews

**FIGURE 2:**
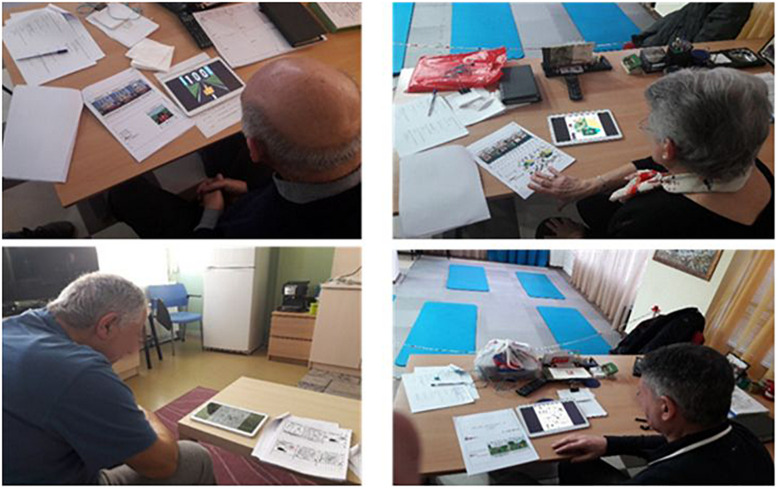
The semi-interview setting in the context of PGS co-design process (Permission and consent have been obtained from the participants to appear on the image).

Ten PD patients and four clinicians were interviewed (from North Greek Parkinson’s Disease Association, Greece), contributing with important feedback/suggestions regarding the 14 PGS game-scenarios. For that purpose, each game-scenario was incorporated in a short video and it was presented to the user in a tablet (see Figure 2). After the visualization of the videos (related with the 14 PGS game-scenarios), a semi-structured interview (∼1 h) was conducted based on different questions (see Supplementary Material). In this way, the participants had an opportunity to be involved as co-designers/creators of the games, when they were asked to re-design the games, giving useful suggestions regarding the enhancement of each game-scenario.

#### Web-Surveys

A set of 104 participants [*mean*±*age* = std ± 49.3 ± 16.3 *years; age range* = [21–83] *years* voluntarily and anonymously responded to the Web-survey (average missing values < 4). Their distribution in different categories (see Part I), was: PD patients (*n*_*1*_ = 40; *mean ± age = std* ± 51.9 ± 15.9 years), Healthcare professionals with experience in PD patients (*n*_*2*_ = 8; *mean age ± std* = 50.2 ± 16 years), Researchers (*n*_*3*_ = 35; *mean age ± std* = 47 ± 16.1 years), Game designer/programmers (*n*_*4*_ = 13; *mean age ± std* = 48.4 ± 16 years), and Participants without PD (n_5_ = 8; *mean age ± std* = 46.2 ± 15 years). The number of responders (104) lies within the acceptable limits, as, usually, in the digital gaming research area where older adults are involved, there are not so many studies with sample size greater than 100 participants (Marston et al., 2016). Adequate variety in the responders’ categories was also noticed, fostering the importance of inclusion of different type of stakeholders in SGs design settings (Emmerich and Bockholt, 2016).

#### Implementation

The GBL-related data (see section “GBL-based Selected Game-Design Factors”) derived from Parts III and IV were subjected to linear regression analysis (Montgomery et al., 2012); for the latter, the IBM SPSS 20 was used^2^. The regression analysis incorporates fitting of linear models, producing relevant fitting statistics, for analyzing multifactor data. The outcome is useful, as the relationship between a variable of interest (the response) and a set of related predictor variables is expressed in closed form, i.e., an equation, grounded on a well-developed statistical theory (Montgomery et al., 2012). Before applying the regression analysis, outliers were handled and the measurement level was adjusted, accordingly.

As it was already mentioned, the transferability of the positive effects of the HCI-SG to real-life settings is the main motivation behind the HCI-SG design. Consequently, here, the *TransferRealLife* variable was considered the variable of interest (dependent/target variable), whereas the rest of the variables (17 in total), directly relating with the examined eight game factors (see section “GBL-based Selected Game-Design Factors”), were considered as independent variables. In this way, via the *TransferRealLife* variable, the potentiality of each HCI-SG to assist the PD patients in using the acquired game skills/experiences in their real-life contexts, as scaffolds to cope with the related PD symptoms, was captured.

## Results and Discussion

### Semi-Interviews Outcomes

Qualitative results derived from semi-structured interviews, revealed that PD patients do not like difficult and sad activities, in general. In addition, some game-scenarios were positively connected with childhood environment of the participants (for this case, retro games could be seen as an alternative scenario to consider in the design of the games). On the other hand, the importance to keep the users’ engagement was also underlined by the PD patients. For instance, regarding the DietaryGames, one participant suggested that the “Sudoku” game could be replaced with memory games format, in order to facilitate the mechanics of the game *à posteriori*. Concerning the real scope/objective of the game, one PD patient revealed that the users must feel that the game is useful for them to engage the user with the game. In this way, the game could be designed, in order to be useful at one domain but give the cause of the perceived usefulness for other domains. In the case of EmoGames, some participants suggested that the faces in the mimic games (and possibly in the “Rhapsody of faces” games) could be photos of patients’ friends; consequently, the users could mimic fun faces of their friends and the difficulty level of each face could be automatic calculated based on the success level of the players.

Clinicians, in general, underlined the positive and important contribution of the PGS research dimension in supporting PD patients. In addition, all clinicians feel that contributed in a positive and significant way to the design of the games, providing useful and constructive feedback and comments.

The main results obtained from this qualitative study were very useful to refine some relevant aspects of the storyboards (e.g., colors—dark colors used in some scenarios were changed; objects—very small objects and very close to each other were improved (e.g., apples, fish), graphics—scenarios using childish graphics were refined) and to help in defining the relevant methodology to be followed for the game-design study that is described in the succeeding subsection. The results comply with the view that the “co-creation” process should be seen as a dynamic and continuous process, starting from the designing and continuing through the development and evaluation phases of the games.

### Web-Survey Outcomes

Statistical, non-parametric analysis of the data resulted from Part II (section “Web-Survey”) showed no statistically significant differences (*p* > 0.05) between the categories of the participants regarding: use of smart devices, preference of SG type, and prior experience in SG use. Hence, all data resulted from Part III (section “Web-Survey”) were handled as a unified dataset to be subjected in regression analysis (section “Experimental Setup and Implementation Issues”).

#### RQ1-Related Results

**FIGURE 3:**
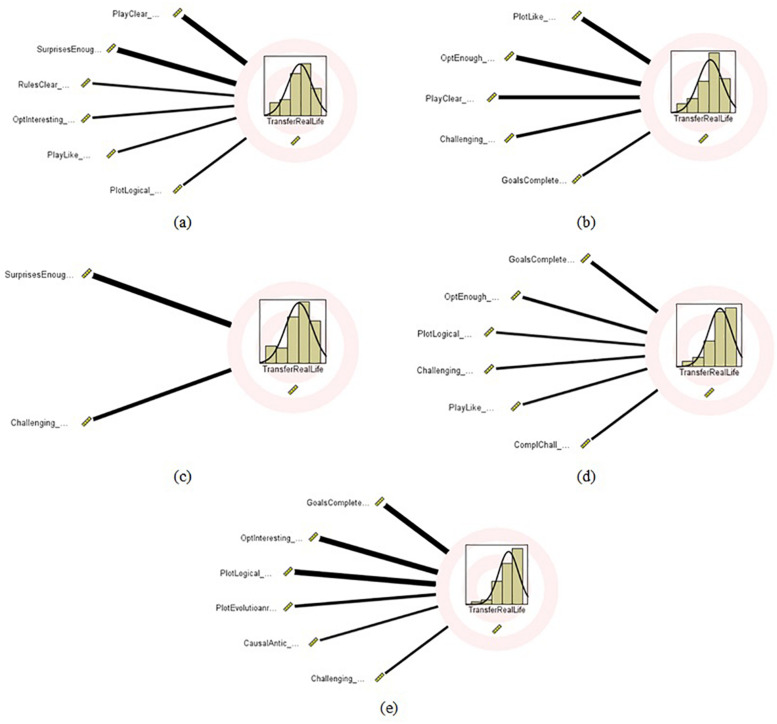
The effect of the significant variables (*p* < 0.05) to the estimated *TratisferRealLife* variable, denoted with the thickness of the connected line for each game category: **(a)** ExerGames, **(b)** DietaryGames, **(c)** EmoGames, **(d)** Hand Writing Games, and **(e)** VoiceGames (the corresponding values are listed in Table 1).

Table 2 presents the contribution of the most important variables (from a total of 17 variables) associated with the eight GBL-based game-design factors that contribute the most to the prediction of the *TransferRealLife* variable. From this table, we can see that for the case of ExerGames, the most effective variables in predicting *TransferRealLife* variable (in a decreasing order) are: the *PlayClear* (denoting that the gameplay is clear) (∼0.36), the *SurprisesEnough* (pointing out that enough surprises exist in the game) (∼0.26), followed by the *RulesClear* (denoting clarity in the game rules) (∼0.10). Similarly, for the DietaryGames, the most important variables (in a decreasing order) are the *PlotLike* (i.e., the user would like to follow the story’s development) (∼0.29), *OptEnough* (i.e., the options of the game are enough) (∼0.28), *PlayClear* (i.e., the gameplay is clear) (∼0.17), followed by the *Challenging* (i.e., the game is challenging) (∼0.14) and *GoalsComplete* (i.e., the responder can complete the game goals) (∼0.12). Accordingly, for the EmoGames, the variable *SurprisesEnough* (∼0.57) and *Challenging* (i.e., the game is challenging) (∼0.20) are the two most important variables. For the HandwritingGames case, the most important variables (in a decreasing order) are: the *Goals Complete* (i.e., the responder can complete the game goals) (∼0.18), *OptEnough* (i.e., the options of the game are enough) (∼0.15), followed by *Plot Logical* (i.e., the game plot is logical) (∼0.10). Finally, for the VoiceGames case, the most important variables (in a decreasing order) are: the *GoalsComplete* (∼0.32), *OptInteresting* (i.e., the game content is plentiful and interesting) (∼0.24), followed by *Plot Logical* (i.e., the game plot is logical) (0.19). Figure 3 shows a visualization of the most important variables per HCI-SG.

**TABLE 2:** The analytical values of the examined parameters to the estimated *TransferRealLife* for the: ExerGames, DietaryGames, EmoGames, Handwriting/VoiceGames.

Source	Sum of squares	*df*	Mean square	*F*	Sig.	Importance
**EXERGAMES**						
*Estimated Model*	198.54	7	28.36	39.41	**0.000**	–
PlayClear	16.19	1	16.19	22.50	**0.000**	0.361
SurprisesEnough	11.79	1	11.79	16.38	**0.000**	0.263
RulesClear	4.83	1	4.83	6.71	**0.010**	0.108
OptInteresting	4.00	1	4.00	5.56	**0.019**	0.089
PlayLike	3.30	1	3.30	4.58	**0.033**	0.073
PlotLogical	3.21	1	3.21	4.46	**0.035**	0.072
*Residual*	213.74	297	0.72			
*Total*	412.28	304				
**DietaryGames**						
*Estimated Model*	177.84	5	35.57	51.79	**0.000**	–
PlayLike	9.69	1	9.69	14.11	**0.000**	0.291
OptEnough	9.15	1	9.15	13.33	**0.000**	0.275
PlayClear	5.75	1	5.75	8.37	**0.004**	0.173
Challenging	4.78	1	4.77	6.95	**0.009**	0.143
GoalsComplete	3.92	1	3.93	5.72	**0.017**	0.118
*Residual*	203.95	297	0.69			
*Total*	381.79	302				
**EmotionalGames**						
*Estimated Model*	126.26	5	25.25	29.80	**0.000**	–
SurprisesEnough	19.21	1	19.21	22.67	**0.000**	0.572
Challenging	6.88	1	6.88	8.12	**0.005**	0.205
*Residual*	166.06	196	0.85			
*Total*	292.32	201				
**HandwritingGames**						
*Estimated Model*	194.19	11	17.65	36.27	**0.000**	–
GoalsComplete	4.80	1	4.80	9.87	**0.002**	0.184
OptEnough	3.84	1	3.84	7.89	**0.005**	0.147
PlotLogical	2.69	1	2.69	5.52	**0.019**	0.103
Challenging	2.54	1	2.54	5.21	**0.023**	0.097
PlayLike	2.13	1	2.13	4.37	**0.038**	0.081
ComplChall	2.05	1	2.05	4.21	**0.041**	0.078
*Residual*	138.72	285	0.49			
*Total*	332.92	296				
**VoiceGames**						
*Estimated Model*	170.03	8	21.25	58.40	**0.000**	–
GoalsComplete	11.17	1	11.17	30.70	**0.000**	0.322
OptInteresting	8.28	1	8.28	22.75	**0.000**	0.238
PlotLogical	6.61	1	6.61	18.15	**0.000**	0.190
PlotEvolucionary	2.56	1	2.56	7.04	**0.008**	0.074
CausalAntic	2.41	1	2.41	6.63	**0.011**	0.069
Challenging	1.61	1	1.61	4.41	**0.036**	0.046
*Residual*	105.90	291	0.36			
*Total*	275.93	299				

**Significant parameters* (*p* < 0.05) are *denoted with bold.**

These findings refine further the selection of the GBL-based game factors, converging the analysis that combined literature review and critical thinking to an efficient game factors selection. Moreover, the interdependencies amongst these game factors are revealed through the regression analysis outcomes.

#### RQ2-Related Results

**FIGURE 4:**
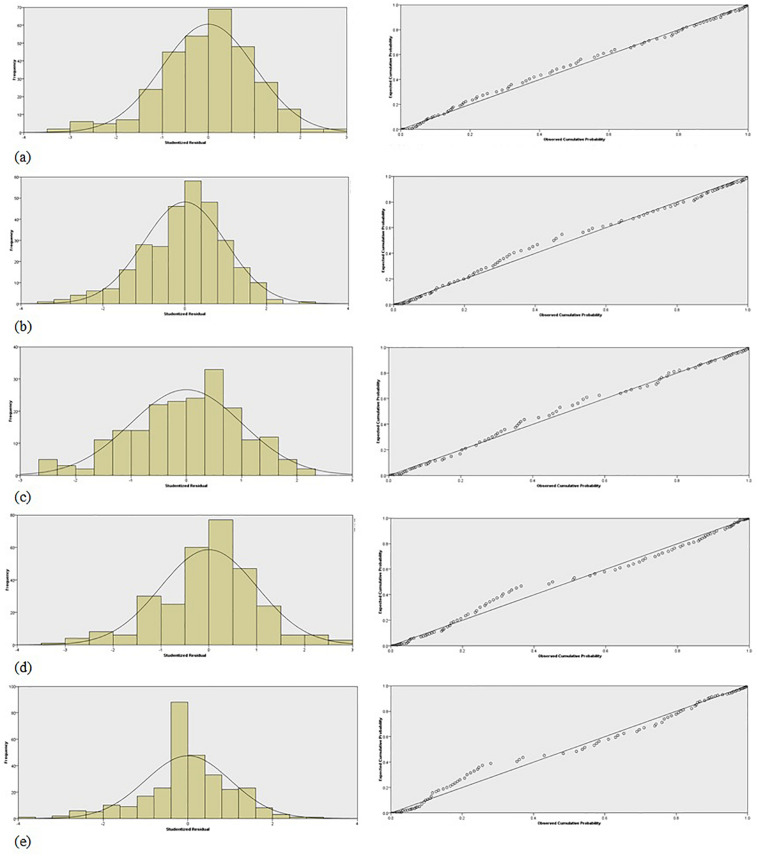
The distribution of the Studentized residuals (bar-plot) of the prediction of the *TransferRealLife* parameter for the: **(a)** ExerGames (*mean* = 0; *std* = 1.005; *N* = 30S); **(b)** DietaryGames (*mean* = 0; *std* = 1.004; *N* = 303); **(c)** EmoGames (*mean* = 0; *std* = 1.007; *N* = 202); **(d)** Hand^TM^ ting Games (*mean* = 0; *std* = 1.011; IV = 297); and **(e)** VoiceGames (mean = 0; std = 1.008; A/ = 300). along with the overlaid normal distribution (solid line) (left panel); and (ii) the observed cumulative probabilities of the Studentized residuals of the prediction of *the TransferRealLife* parameter versus the expected ones (circles) for the: **(a)** ExerGames. **(b)** DietaryGames. **(c)** EmoGames. **(d)** Handwriting Games, and **(e)** VoiceGames; the diagonal line represents the normal distribution (solid line) (right panel).

Figure 4 illustrates the prediction residuals of the predictive performance of the regression analysis. More specifically, the distribution of the Studentized residuals of the prediction of the *TransferRealLife* variable (left column), combined with the superimposed normal distribution (solid line) (right column), are depicted for each game category: (A) ExerGames, (B) DietaryGames, (C) EmoGames, (D) HandwritingGames, and (E) VoiceGames.

Moreover, in Figure 4 (right column) the observed cumulative probabilities of the Studentized residuals of the prediction of the *TransferRealLife* variable versus the expected ones (circles) are depicted (normal distribution (solid line) is represented by the diagonal). Figure 4 justifies the reliable performance of the adopted regression analysis, i.e., the model inference (confidence intervals, model predictions) are valid, as the estimated residuals followed the assumed Gaussian distribution (*i*.*e*.,*mean* = 0.0. and *std* = 1.005; *mean* = 0.0 and *std* = 1.004; *mean* = 0.0 and *std* = 1.007; *mean* = 0.0 and *std* = 1.011; *mean* = 0.0 and *std* = 1.008).

Estimated regression model coefficients, accompanied by their statistical significance values, are shown in Table 3.

**TABLE 3:** The analyzed parameters along with their importance for the estimation of the *TransferRealLife* parameter for the: ExerGames, DietaryGames, EmoGames, Handwriting/VoiceGames.

Model term	Coefficient	Std error	*t*	Sig.	95%confidence interval	
					Lower	Upper
**ExerGames**						
PlayClear	0.426	0.09	4.74	**0.000**	0.249	0.602
SurprisesEnough	0.224	0.06	4.05	**0.000**	0.115	0.333
RulesClear	−0.247	0.10	−2.59	**0.010**	−0.435	−0.059
OptInteresting	0.147	0.06	2.36	**0.019**	0.024	0.270
PlayLike	0.131	0.06	2.14	**0.033**	0.011	0.252
PlotLogical	0.137	0.07	2.11	**0.035**	0.009	0.264
**DietaryGames**						
Intercept (β_0_)	0.514	0.22	2.34	**0.020**	0.082	0.945
PlayLike	0.222	0.06	3.76	**0.000**	0.106	0.338
OptEnough	0.212	0.06	3.65	**0.000**	0.098	0.326
PlayClear	0.162	0.06	2.89	**0.004**	0.052	0.272
Challenging	0.136	0.05	2.64	**0.009**	0.035	0.238
GoalsComplete	0.142	0.06	2.39	**0.017**	0.025	0.259
**EmotionalGames**						
Intercept (β_0_)	0.827	0.35	2.34	**0.020**	0.131	1.522
SurprisesEnough	0.364	0.08	4.76	**0.000**	0.213	0.514
Challenging	0.193	0.07	2.85	**0.005**	0.059	0.326
**HandwritingGames**						
GoalsComplete	0.209	0.07	3.14	**0.002**	0.078	0.339
OptEnough	0.164	0.06	2.81	**0.005**	0.049	0.280
PlotLogical	0.169	0.07	2.35	**0.019**	0.027	0.311
Challenging	0.124	0.05	2.28	**0.023**	0.017	0.231
PlayLike	−0.135	0.06	−2.09	**0.038**	−0.262	−0.008
ComplChall	−0.119	0.06	−2.05	**0.041**	−0.232	−0.005
**Voice Games**						
GoalsComplete	0.279	0.05	5.54	**0.000**	0.180	0.378
OptInteresting	0.262	0.06	4.77	**0.000**	0.154	0.370
PlotLogical	0.233	0.06	4.26	**0.000**	0.126	0.341
PlotEvolucionary	−0.136	0.05	−2.65	**0.008**	−0.237	−0.035
CausalAntic	0.138	0.05	2.57	**0.011**	0.033	0.244
Challenging	0.095	0.05	2.10	**0.036**	0.006	0.184

**The significant parameters* (*p* < 0.05*) are denoted with bold and are those that are only used for the corresponding regression equations (1)–(5).**

For the ExerGames case, the *PlayClear, SurprisesEnough*, *RulesClear*, *OptInteresting*, *PlayLike*, and *PlotLogical* variables exhibit statistical significance; hence, are involved in a valid regression equation (see also Figure 3), formed as follows:

TransferRealLife=  0.426⋅PlayClear+0.224⋅

S⁢u⁢r⁢p⁢r⁢i⁢s⁢e⁢s⁢E⁢n⁢o⁢u⁢g⁢h-0.247⋅R⁢u⁢l⁢e⁢s⁢C⁢l⁢e⁢a⁢r+0.147⋅O⁢p⁢t⁢I⁢n⁢t⁢e⁢r⁢e⁢s⁢t⁢i⁢n⁢g

(1) +0.131⋅P⁢l⁢a⁢y⁢L⁢i⁢k⁢e+0.137⋅P⁢l⁢o⁢t⁢L⁢o⁢g⁢i⁢c⁢a⁢l+ε,

where ε (epsilon) is a random zero-mean error component expressing the distance (above or below) the true regression line (i.e., the line of means) the actual observation lies (ε notation is similarly used across all regression equations that follow).

For the case of the DietaryGames, the *PlayLike*, *OptEnough*, *PlayClear*, *Challenging*, and *GoalsComplete* variables, along with the *Intercept* (β_0_), show statistical significance; hence, are incorporated in a valid regression equation (see also Figure 3), formed as below:

TransferRealLife=  0.514+0.222⋅

PlayLike+0.212⋅OptEnough+0.162⋅PlayClear+0.136⋅

(2) C⁢h⁢a⁢l⁢l⁢e⁢n⁢g⁢i⁢n⁢g+0.142⋅G⁢o⁢a⁢l⁢s⁢C⁢o⁢m⁢p⁢l⁢e⁢t⁢e+ε.

In the EmoGames, the *SurprisesEnough* and *Challenging* variables, along with the *Intercept* (β_0_), exhibit statistical significance; hence, are involved in a valid regression equation (see also Figure 3), formed as follows:

(3) TransferRealLife=0.827+0.364⋅S⁢u⁢r⁢p⁢r⁢i⁢s⁢e⁢s⁢E⁢n⁢o⁢u⁢g⁢h+0.193⋅C⁢h⁢a⁢l⁢l⁢e⁢n⁢g⁢i⁢n⁢g+ε.

For the HandwritingGames, the *GoalsComplete*, *OptEnough*, *PlotLogical*, *Challenging*, *PlayLike*, and *ComplChall* variables show statistical significance; hence, are involved in a valid regression eqnarray (see also Figure 3), formed as below:

TransferRealLife=0.209⋅GoalsComplete+0.164⋅

O⁢p⁢t⁢E⁢n⁢o⁢u⁢g⁢h+0.169⋅P⁢l⁢o⁢t⁢L⁢o⁢g⁢i⁢c⁢a⁢l+0.124⋅C⁢h⁢a⁢l⁢l⁢e⁢n⁢g⁢i⁢n⁢g-

(4) 0.135⋅P⁢l⁢a⁢y⁢L⁢i⁢k⁢e-0.119⋅C⁢o⁢m⁢p⁢l⁢C⁢h⁢a⁢l⁢l+ε.

Finally, in the case of the VoiceGames, the *GoalsComplete*, *OptInteresting*, *PlotLogical*, *PlotEvolucionary*, *CausalAntic*, and *Challenging* variables show statistical significance; hence, are incorporated in a valid regression equation (see also Figure 3), formed as below:

TransferRealLife=0.279⋅GoalsComplete+0.262⋅

OptInteresting+0.233⋅PlotLogical-0.136⋅

⁢P⁢l⁢o⁢t⁢E⁢v⁢o⁢l⁢u⁢c⁢i⁢o⁢n⁢a⁢r⁢y+0.138⋅C⁢a⁢u⁢s⁢a⁢l⁢A⁢n⁢t⁢i⁢c

(5) +0.095⋅C⁢h⁢a⁢l⁢l⁢e⁢n⁢g⁢i⁢n⁢g+ε.

As an example, from (3) it is clear that when one-point in *SurprisesEnough* variable is increased results in a corresponding increase of 0.364 points solely in the *TransferRealLife* variable, when the rest ones remain constant. Similarly, for the case of one-point increase in *Challenging* variable results in a corresponding increase of 0.193 points in the *TransferRealLife* variable, if all other variables remain constant. The same analysis can be followed for all the rest of the equations and corresponding game category.

#### RQ3-Related Results

The knowledge deriving from the RQ1/RQ2-related results is used to form some recommendations to PD-related HCI-SGs designers (addressing RQ3). However, it should be underlined that not all GBL-based game-design factors affect the transferability of the PD-related game experience to a real-life context in the same way. Apparently, the PD-related HCI-SG design is significantly dependent on the gameplay and its clear definition. In this context, it is noteworthy that when players are asked to describe and judge a game, they sometimes analyze “what the game is about,” thus talking about the game context. In these cases, they usually focus especially on “what you have to do,” i.e., the goals of the game, thus display more interest for functional aspects than for aesthetic aspects of the context. However, more often the focus of players’ analysis is set on the “what you can do” factor, i.e., the gameplay of the game. In many cases, players neglect the context and even the very same goals of the game, to focus on the gameplay activities that may be carried out in order to win. Hence, gameplay is the primary focus of players’ attention when it comes to judging a game. Even more, according to players’ opinions, flaws in functional elements of a game cannot be balanced by any non-functional aspect of the design, since a very good game context cannot sustain motivation if gameplay activities are ill-designed (Fabricatore et al., 2002). All these point out the relevance of the gameplay, leading us to consider it as a cornerstone in the game design.

Another important game factor relates with the number of surprises in the game play. If they are enough, they could be used as triggers to the user’s game engagement; hence, influencing the way all interactions within the game, such as balanced body movements, body reaction time, could be transformed to everyday behaviors, easing coping with the PD symptoms (e.g., movement and balance issues, like rigidity, limited range of motion, balance and coordination issues, abnormal posture). Taking this further and within the GBL context, game surprises could also generate manageable cognitive conflicts that can stimulate players to engage more in the processes like efficient knowledge organization and integration; these, actually, boost learning and behavioral change, without, in fact, jeopardizing the motivational appeal of the game (Wouters et al., 2017). Being a disruptor of an active expectation, game surprise can provoke emotional reaction, combined with cognitive goal setting, directing the attention to possible explanations about the occurrence of the surprise *per se*; thus, fostering learning further (Foster and Keane, 2015), re-educating, training and informing PD patients.

### Practical Implications

For end-users to reach the desired positive effect from HCI-SGs, the latter need to integrate a supportive system that merges multidisciplinary fields. It still remains an open problem to all game stakeholders (i.e., designers, developers, researchers) how the design and development processes have to be organized to ensure that all different aspects of the game are properly addressed (Braad et al., 2016). In order to address this issue, the present study explores the practical and direct implications of its findings on the design/development processes of HCI-SGs within the i-PROGNOSIS PGS framework. More specifically, the most important game factors identified here can be used by the game designers in many iterations of the design process and be re-adjusted in a dynamic way (when and where it is required). This refinement in the design process could further optimize the developmental phase, in terms of increased quality of the HCI-SG, maximizing the positive impact to PD patients.

It should be noted that effective HCI-SG is not only contingent upon sound instructional design, but also closely tied to the creation of a successful entertainment game. In other words, play and entertainment should not be secondary in HCI-SGs. This conclusion is in line with existing theoretical approaches on HCI-SGs, including those works which have touted the utility of endogeny (i.e., tying game interactions to learning content) (Squire, 2006), the benefits of curiosity and exploration of game content (Malone, 1980), and the overall motivational benefits of gameplay (Sweetser and Wyeth, 2005). It is the great paradox of HCI-SGs that these games are simultaneously praised for their learning benefits but reduced in “game-ness” in order to appear more valid (Pavlas, 2010). Apparently, HCI-SGs are enhanced by the very thing that makes them games: play.

Indeed, one of the defining features separating HCI-SGs from simulations is their reliance upon play interactions (Baranauskas et al., 1999). Moreover, the DIN SPEC 91380 Serious Games Metadata Format was considered in the present work, including the quality criteria for both the serious (e.g., game goals, methods/feedback, rewards, quality, sustained effects) and the games (e.g., user enjoyment, flow, user engagement, media presentation, graphics, background music) parts, and mostly the balance between them (e.g., scientific foundation, appropriate interaction technology) (Caserman et al., 2020). In this way, the metadata format gives an opportunity to provide a technical framework able to describe and select the most appropriate games to match the needs of the users (Göbel and Maddison, 2017).

#### Game-Based Learning and Game-Based Assessment

The findings presented so far can be further been seen from a combined view of both the GBL principle and the integration of Game-based Assessment (GBA) approach. This could further contribute to the game design improvement by using interdisciplinary approaches and methodologies that allow to examine cognitive, emotional, and motivational processes during gameplay to better understand the quality of interaction of the users/players, providing more than pre-post-test measures/self-reports (Mayer, 2014; Taub et al., 2017). This can be achieved via the employment of targeted assessment metrics that are associated with biosignals, such as Heart Rate (HR), Heart Rate Variability (HRV), Breathing Rate (BR), and/or body postures/movements (e.g., gait, body gestures, fingers fine movements), and/or sounds (e.g., voice, breathing sounds), and/or emotions (e.g., via facial expressions). Some characteristics examples listed below.

• *PGS ExerGames Design Approach:* Concerning the PGS ExerGames design approach, with the integration of indicators informed by HR, HRV, BR data when using depth sensors or wearables (such as smartwatch/smartbands), the game difficulty of each Exergame can be regulated to become higher (lower) when players’ heart rate falls below (exceeds) predefined thresholds based on players’ individual baseline heart rate. This can allow better regulation of the cardiovascular functionality during the gaming, as is exemplified in the work of Villafaina et al. (2020), where they show a significant effect of an Exergame-based intervention on the HRV as a therapy in patients with fibromyalgia.
In a similar view, behavioral information captured from body expression can be directly connected to the HCI-SG dynamic control. In fact, several body tracking systems have been employed in the health environments. For instance, the Microsoft Kinect^®^ sensor have been used for neurological rehabilitation (Knippenberg et al., 2017) and for analyzing PD posture and lower limb tasks (Ferraris et al., 2019). In the same line, the use of the MentorAge^®^ sensor tracking system has proven its capabilities in real life environments (e.g., Anzivino et al., 2018; Petsani et al., 2019; Dias et al., 2020). In this vein, gait features can be extracted by relevant ExerGames that request from the user to move a couple of steps (within the depth camera range) toward different directions. In a similar scenario, using appropriately designed gameplay, the patients can be instructed to progressively reach out large amplitude movements (e.g., hand grasping fruits at different heights from a virtual tree); based on the tracking of the patients’ performance (e.g., maximum angle, speed, articulation), using a depth camera, a personalized set of intermediate goals can be defined just above each patient’s average performance. Clearly, this can be related to various symptoms of PD, such as difficulty in gait, balance and coordination, and be reflected to the in-game metrics and mechanisms of the relevant ExerGames (Dias et al., 2020).
• *PGS DietaryGames design approach:* For capturing the eating behavior, the Mandometer^®^ personal scale (with a chewing sensor) can be used to measure the meal mechanics, representing a weighting scale that counts the progressive weight changes from a plate with food during a meal. The Mandometer^®^ system, includes a portable computer connected to a scale, and can be used to help patients with eating disorders to normalize their eating behavior; for instance, during the course of a meal, the system displays continuous feedback to the patient concerning the consumed amount and eating speed. In addition, some studies have investigated the effect of eating rate upon food intake quantity using the Mandometer^®^ personal scale as an automated means to analyze food intake, providing feedback based on food weight change over time (Ioakimidis et al., 2009).
From these perspectives, a Mandometer^®^ scale can be combined with the PGS DietaryGames that simulate the eating gestures to measure the meal mechanics with potentiality to monitor progressive weight changes from a plate, in order to explore different dietary scenarios, helping health professionals to provide prognostic simulations for diseases effect (such as PD) on dietary habits.
• *PGS Handwriting/Voice Games design approach:* Another form of GBA information relates with the movements of the fingers. Some HCI-SGs have focused on predictive PD analytics through pad games based on the data collected regarding the trails of the player’s fingers sliding on the screen (Liu et al., 2013); through this game, real-time data analysis is performed and alerts can be sent to the doctors in case of detection of high risk of having PD. The PGS HandwritingGames approach, in particular, targets on enhancing the handwriting patterns of early PD patients by prompting to write/draw specific letters and numbers with guidance lines and within specific space limits, while providing real-time feedback of the performance. Grids and lines appear and adapt correspondingly to the user’s writing profile and micrographia level. In this way, bradykinesia and rigidity PD symptoms can be reflected in the in-game metrics, enhancing the GBA potentialities of the game.
Voice-based GBA information is another form of HCI-SG contribution to the capturing (e.g., by the tablet’s or smartphone’s microphone) and supporting human behavior expression and health status. Features, such as volume, loudness, pitch, formant frequencies can be extracted by signal processing algorithms from the patient’s voice during gaming. For example, a digital game to support voice treatment for PD was developed by Krause et al. (2013), where the users had to break enough items, by using their voice, to reach a high score; the results indicated an increased peak voice loudness of the players’ voice when playing the game. Moreover, the design and implementation of a rehabilitation software for dysphonic patients was proposed, exploring the extraction of pitch (Mel) feature from patients’ voice for evaluating the long-time rehabilitation progress (Lv et al., 2015). The PGS VoiceGames approach stimulates the player to pronounce specific vowels/words at constant or alternating voice intensity levels, use shorter sentences and train vocal cords toward hypophonia limitation, a characteristic of PD patients. In this way, the H/V Games enrich the data with their in-game metrics, in order to serve as a “software as a sensor,” toward early PD detection by multimodal monitoring.
• *PGS EmoGames design approach:* The interdependency between emotions and learning suggests an important opportunity for physiological sensor use in GBA approach (Novak and Johnson, 2012; Ninaus et al., 2017). Several studies have used different sensor technologies to determine emotional states (Mandryk and Atkins, 2007); one approach is based on the recording participants’ faces to manually or automatically classify different emotional facial expressions (Littlewort et al., 2011). These approaches are usually based on using facial action units from the Facial Action Coding System (Cohn et al., 2007).
From this perspective, new ways to detect motor impairments in facial muscles by analyzing images/photos (selfies) and/or videos that are captured by the user, using e.g., his/her tablet/smartphone front-camera, can be considered in the design and development of the EmoGames approach. For instance, hypomimia detection methods may be extended to use videos from standard cameras (e.g., mobile phone) instead of a 3-D camera sensor or other more obtrusive sensors (Vinokurov et al., 2015).

This dynamic regulation of the HCI-SG using the aforementioned GBA information sources provides personalization and increase in the importance of the human health status as a regulation factor during the user interaction, lying within the emerging field of Physiological Computing (PC), and makes the HCI-SG capable of sensing, processing, reacting and interfacing the digital and analog worlds.

#### Limitations and Future Work

Clearly, the analysis presented here could be further expanded by employing different parameter sets and/or other predictors grounded on different theoretical approaches. An example includes expansion of the regression analysis on between constructs to simultaneous examination of the entire model subsets, incorporating structural equation modeling. This could shed light in the model appropriateness. Furthermore, evaluation of the development of the HCI-SGs using the Technology Acceptance Model (TAM) (Legris et al., 2003) could be employed, constructing a holistic approach in the HCI-SGs design and evaluation. Clearly, this integrated approach could maximize the benefit to and acceptability from PD patients. In this way, a new design model with the corresponding regression equations across the i-PROGNOSIS PGS can be formed, enabling a macroscopic level of analysis for the successful prediction of the transferability of the PD-related games to the real-life context. For that purpose, the collection of constructive feedback from PD patients/medical experts allow their involvement as co-creators of the HCI-SGs design. In addition, next steps will also consider the user acceptance evaluation to obtain feedback from real-users, in order to ensure that the i-PROGNOSIS PGS mobile application is easy-to-use, requiring smooth assistance for its use, and serving for the technical refinement of the i-PROGNOSIS PGS mobile application.

Finally, based on Artificial Intelligence (AI)-Assisted game design recent perspectives (Liapis et al., 2019), a seamless integration of the AI concept within the game design is foreseen, including adaptation algorithms that have the capability to change/re-adapt the games and the different levels of difficulty, according to the PD patient’s needs and his/her social engagement, as well as the development of the personalized Avatar authoring tool. This, actually, reinforces the role of HCI-SGs within the PC context, supporting further the design, implementation, and evaluation of next-generation gamified human-computer interfaces.

## Conclusion

An exploration about the game design elements that play important role in assistive HCI-SGs for PD patients was presented here. In particular, the GBL and GBA design frameworks were explored and the main game-design parameters from the i-PROGNOSIS PGS were identified and discussed based on qualitative data from semi-structured interviews and data from a relevant Web-survey. An adapted GBL framework was identified, based on linear regression data analysis, incorporating the most significant game-design factors that efficiently predict the transferability of the ExerGames, DietaryGames, EmoGames, and Handwriting/Voice Games beneficial effect to real-life context. Finally, extended implications within the context of physiological computing were explored and viewed via the i-PROGNOSIS PGS. The findings reported here can assist game designers to focus on the use of the most significant game-design factors of HCI-SGs in order to sustain and/or improve the quality of everyday living of PD patients.

## Data Availability Statement

The raw data supporting the conclusions of this article will be made available to any qualified researcher upon reasonable request directed to the corresponding author.

## Ethics Statement

All the experimental and ethical protocols were approved by the Bioethics Committee of the Aristotle University of Thessaloniki (AUTH) Medical School, Thessaloniki, Greece (401/31.01.2018), Ethik-Kommission an der Technischen Universität Dresden, Dresden, Germany (EK 451112017), and United Kingdom London—Surrey Borders Research Ethics Committee (18/LO/0074). The patients/participants provided their written informed consent to participate in this study.

## Author Contributions

All authors contributed for the conceptual design of the i-PROGNOSIS Personalized Game Suite. SB and LH analyzed the data. All authors discussed the results and contributed to the manuscript. All authors contributed to the article and approved the submitted version.

## Conflict of Interest

Authors Hugo Silva and Gonçalo Telo were employed by the company PLUX, Wireless Biosignals. Moreover, Konstantinos Filis, Elina Theodoropoulou, and George Lyberopoulos were employed by the company COSMOTE Kinites Tilepekoinonies AE. The remaining authors declare that the research was conducted in the absence of any commercial or financial relationships that could be construed as a potential conflict of interest.

## Footnotes

1 www.storyboardthat.com/

2 https://www.ibm.com/analytics/data-science/predictive-analytics/spss-statistical-software
