# Supplementary figures and images for: Assistive HCI-Serious Games Co-design Insights: The Case Study of i-PROGNOSIS Personalized Game Suite for Parkinson’s Disease
Source: Front Psychol. 2021 Jan 15;11:612835. doi: 10.3389/fpsyg.2020.612835 (PMC7843389; doi:10.3389/fpsyg.2020.612835)

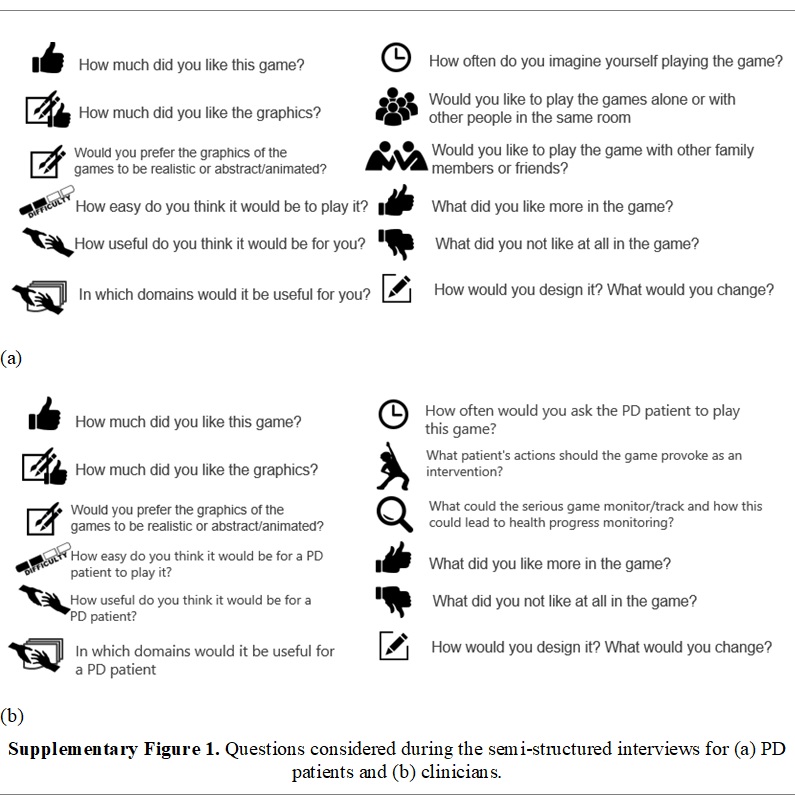

Supplement: Supplementary file 1 [file Image_1.JPEG]

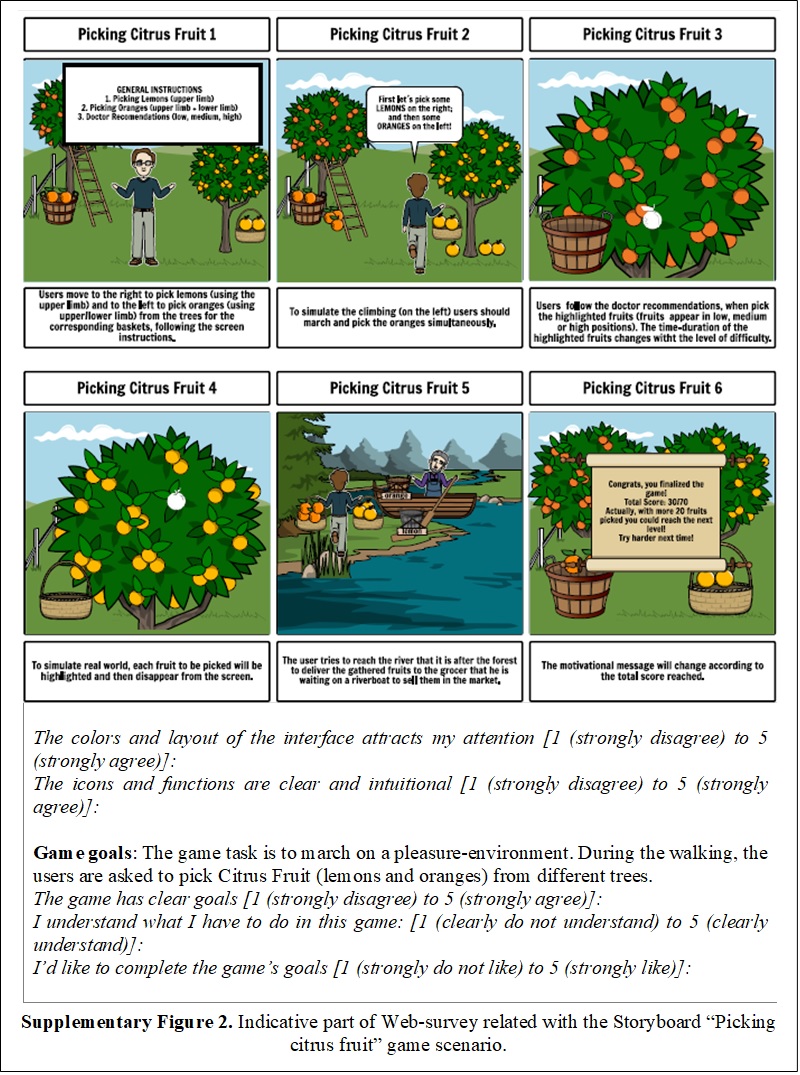

Supplement: Supplementary file 2 [file Image_2.JPEG]

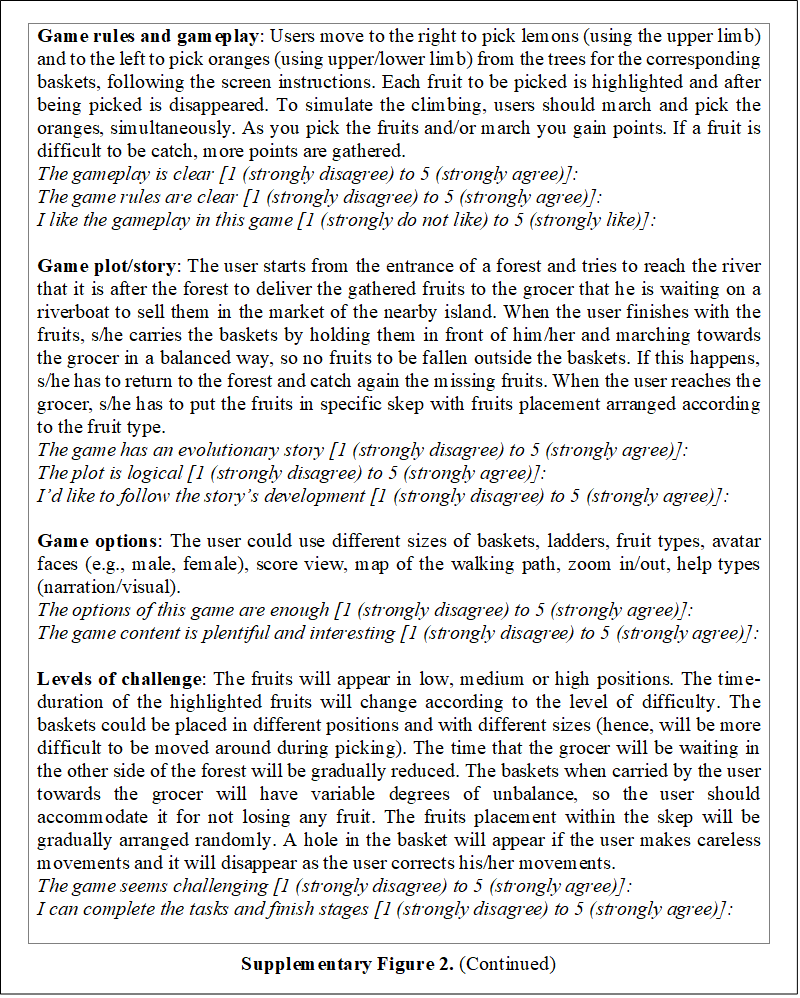

Supplement: Supplementary file 3 [file Image_3.JPEG]

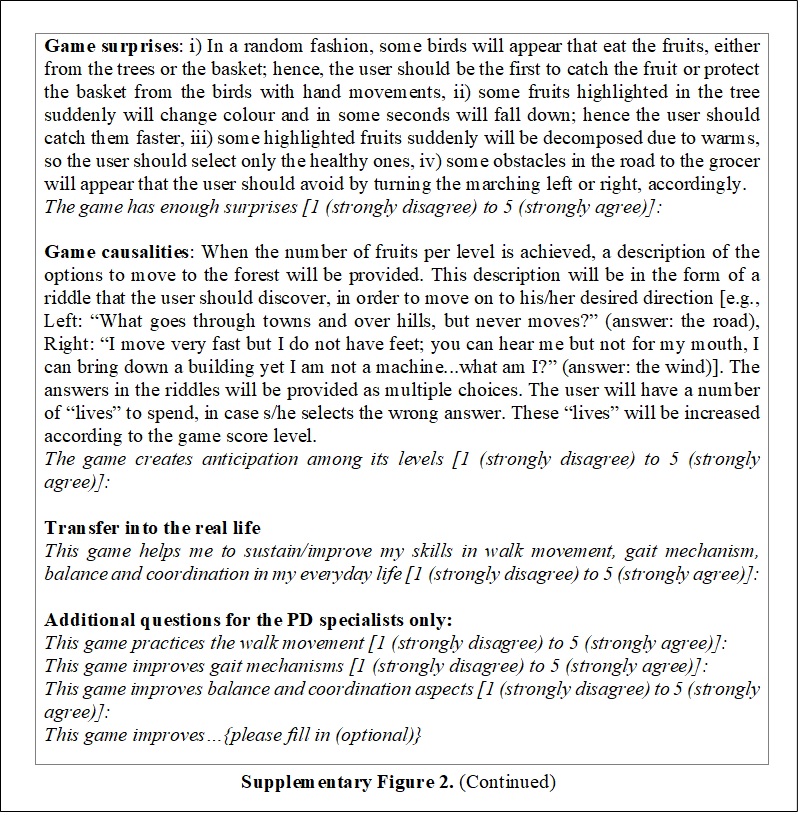

Supplement: Supplementary file 4 [file Image_4.JPEG]
